# Characterization of Sleep in Zebrafish and Insomnia in Hypocretin Receptor Mutants

**DOI:** 10.1371/journal.pbio.0050277

**Published:** 2007-10-16

**Authors:** Tohei Yokogawa, Wilfredo Marin, Juliette Faraco, Guillaume Pézeron, Lior Appelbaum, Jian Zhang, Frédéric Rosa, Philippe Mourrain, Emmanuel Mignot

**Affiliations:** 1 Howard Hughes Medical Institute, Stanford University, Palo Alto, California, United States of America; 2 Stanford Center for Narcolepsy, Stanford University, Palo Alto, California, United States of America; 3 Ecole Normale Supérieure, Paris, France; 4 INSERM Unité 784, Paris, France; Wellcome Trust Sanger Institute, United Kingdom

## Abstract

Sleep is a fundamental biological process conserved across the animal kingdom. The study of how sleep regulatory networks are conserved is needed to better understand sleep across evolution. We present a detailed description of a sleep state in adult zebrafish characterized by reversible periods of immobility, increased arousal threshold, and place preference. Rest deprivation using gentle electrical stimulation is followed by a sleep rebound, indicating homeostatic regulation. In contrast to mammals and similarly to birds, light suppresses sleep in zebrafish, with no evidence for a sleep rebound. We also identify a null mutation in the sole receptor for the wake-promoting neuropeptide hypocretin (orexin) in zebrafish. Fish lacking this receptor demonstrate short and fragmented sleep in the dark, in striking contrast to the excessive sleepiness and cataplexy of narcolepsy in mammals. Consistent with this observation, we find that the hypocretin receptor does not colocalize with known major wake-promoting monoaminergic and cholinergic cell groups in the zebrafish. Instead, it colocalizes with large populations of GABAergic neurons, including a subpopulation of Adra2a-positive GABAergic cells in the anterior hypothalamic area, neurons that could assume a sleep modulatory role. Our study validates the use of zebrafish for the study of sleep and indicates molecular diversity in sleep regulatory networks across vertebrates.

## Author Summary

Sleep disorders are common and poorly understood. Further, how and why the brain generates sleep is the object of intense speculations. In this study, we demonstrate that a bony fish used for genetic studies sleeps and that a molecule, hypocretin, involved in causing narcolepsy, is conserved. In humans, narcolepsy is a sleep disorder associated with sleepiness, abnormal dreaming, and paralysis and insomnia. We generated a mutant fish in which the hypocretin system was disrupted. Intriguingly, this fish sleep mutant does not display sleepiness or paralysis but has a 30% reduction of its sleep time at night and a 60% decrease in sleep bout length compared with non-mutant fish. We also studied the relationships between the hypocretin system and other sleep regulatory brain systems in zebrafish and found differences in expression patterns in the brain that may explain the differences in behavior. Our study illustrates how a sleep regulatory system may have evolved across vertebrate phylogeny. Zebrafish, a powerful genetic model that has the advantage of transparency to study neuronal networks in vivo, can be used to study sleep.

## Abstract 3

Zebrafish sleep, and have the receptor for the wake-inducing molecule hypocretin. While mutation in this receptor causes narcolepsy in mammals, in fish, sleep is fragmented, demonstrating differences in sleep control in vertebrates.

## Introduction

The function of sleep is disputed, with hypotheses ranging from energy conservation to synaptic remodeling and memory consolidation, with the possibility of disparate functions across evolution [1,2]. One approach to this question is to study sleep and sleep regulatory networks in organisms amendable to molecular, neuronatomical, and genetic studies [3–5]. Using purely behavioral criteria, a sleep-like state has been demonstrated in non-mammalian species [6,7]. A sleep-like state has been characterized in Drosophila melanogaster [4], initially using behavioral criteria, and more recently through electrophysiological studies [8]. Identification and characterization of sleep mutants is ongoing [5]. While unquestionably a superb genetic model organism, the phylogenetic distance between *Drosophila* and mammals has produced notable and relevant divergences in usage of neuromodulators. Whereas tyramine and octopamine are critically important in *Drosophila,* they are trace amines of unknown function in mammals and other vertebrates [9]. Further, certain other neurotransmitter systems have not been identified in *Drosophila,* including hypocretin/orexin, an important sleep modulator.

Hypocretins [10], also called orexins [11], are neuropeptides of primary interest in the study of sleep. Indeed, they compose the only neurochemical system known to be involved in the generation of a clear human sleep disorder phenotype, narcolepsy [12–17]. In mammals, preprohypocretin is primarily expressed by neurons of the posterior hypothalamus, with widespread projections to the brain and spinal cord [18]. Two closely related receptors are known, with complementary neuronatomical distribution [11,19]. Of notable importance for sleep regulation, mammalian hypocretin neurons also project widely and activate monoaminergic cell groups, which are generally known to be wake-active (adrenergic, dopaminergic, serotoninergic, and histaminergic) [20–23]. Further, they also project and activate cholinergic cell groups [24–26] important for the regulation of wakefulness and rapid eye movement (REM) sleep. Intracerebroventricular (icv) injections of hypocretin are potently wake-promoting and increase locomotion in mammals [27–29], an effect partially blocked by histaminergic [28] and dopaminergic [30] antagonists, with higher doses inducing stereotypies similar to those observed after massive dopaminergic stimulation [30]. These experiments suggest that hypocretin is a major sleep modulator, and that much of its activity is mediated through direct stimulation of aminergic systems.

Narcolepsy can be observed in multiple mammalian species. In humans, the disorder is primarily due to hypocretin cell loss [14,16]. It is characterized by excessive daytime sleepiness, disturbed nocturnal sleep (inability to stay asleep at night), cataplexy (transient muscle paralysis triggered by emotions), sleep paralysis (transient muscle paralysis during sleep–wake transitions), and hypnagogic hallucinations (dream-like experiences at sleep onset) [31,32]. Many of the symptoms of narcolepsy are related to an abnormal tendency to enter REM sleep rapidly, and to express abnormal REM sleep features while awake (e.g., muscle paralysis) or dreaming (hypnagogic hallucinations). Contrary to popular belief, narcolepsy is not characterized by increased total sleep time over the 24-h period [33,34]. Rather, patients with narcolepsy are unable to consolidate either wakefulness or sleep [33,35].

In dogs, narcolepsy has been studied for over 20 years and has been shown to display all the symptoms of human narcolepsy [36]. It can result from mutations in the hypocretin receptor-2 (HCRTR2) gene (familial cases) [12] or from hypocretin deficiency (sporadic narcolepsy) [37]. In rats and mice that have been engineered to lack hypocretin, hypocretin cells, or HCRTR2, narcolepsy manifests as sleep and wake fragmentation during the day and the night, sudden episodes of muscle weakness, and rapid transitions from wake to REM sleep [15,38,39]. In contrast, hypocretin receptor-1 (HCRTR1) knockout mice do not display sudden episodes of muscle weakness, but have sleep abnormalities [39,40]. These comparisons highlight the similarity of the phenotype across mammalian species and a primary role for HCRTR2-mediated transmission.

The zebrafish is a powerful genetic model that has, as a vertebrate, the advantage of sharing a similar central nervous system organization with mammals [41,42]. We, and others, have shown that principal actors of sleep regulation in mammals are largely conserved in zebrafish, including monoaminergic [43–46], cholinergic [47], and hypocretinergic (orexin) [48–50] cell groups. In addition to conservation of cell groups, responses to various hypnotic drugs such as H1 histaminergic antagonists, melatonin agonists, alpha-2 adrenergic agonists, and GABAergic modulators are also conserved in the zebrafish [51–53]. A sleep-like state was first characterized in 7- to 14-d-old zebrafish larvae by Zhdanova and colleagues, who demonstrated an elevated arousal threshold, a reduced breathing respiratory rate, and a compensatory rest rebound in response to deprivation as well as modulation by hypnotic drugs [51,54]. More recent studies have also explored sleep architecture in zebrafish larvae [50].

These findings raise the question of whether conserved neuromodulators function in the same roles to regulate sleep in fish as in mammals, or whether there are presently unappreciated divergences. The study of fish sleep is also interesting because the fish is a cold-blooded vertebrate, and mammalian-like sleep architecture, as defined by REM sleep and non-REM sleep, typically emerged with homeothermy [55]. To address these questions, we performed a detailed characterization of sleep in adult zebrafish and characterized sleep abnormalities in adult zebrafish lacking the sole hypocretin receptor.

## Results

### Defining Sleep in Adult Zebrafish

**Figure 1:**
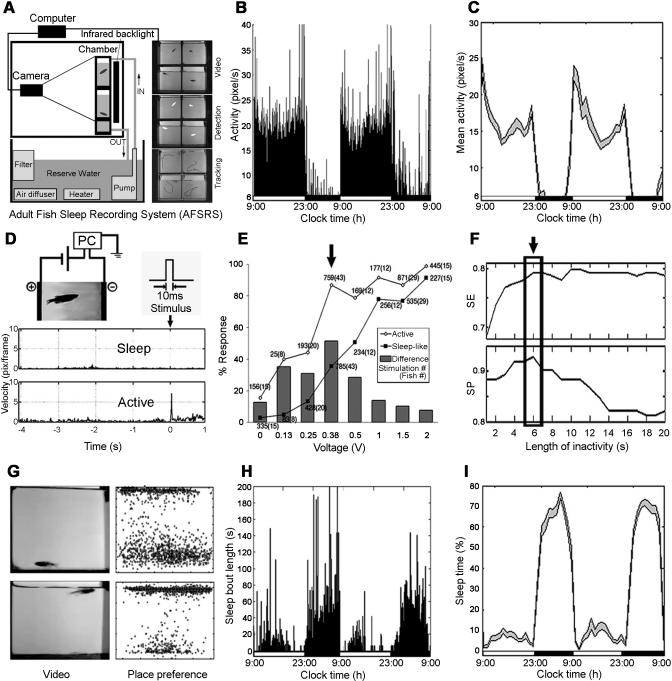
Characterization of Rest in Zebrafish as a Sleep-Like State (A) AFSRS. Images are recorded through backlit chambers using an infrared video camera (30 frames/s), and software then detects and tracks the fish and its movements (see Materials and Methods). See also Video S5. (B) Activity of a single fish displayed as pixels/s for each minute over 2 d with 14 h: 10 h light/dark conditions. Note alternation of periods of activity and inactivity. (C) Mean activity of 13 normal adult fish across the light/dark cycle (± standard error of the mean, represented as shaded areas). Data are plotted as the mean of 2-h blocks, after a 3-d habituation period. (D) Arousal threshold and electrical stimulation experiments. The upper panel shows the electrical stimulation apparatus that applies stimuli through stainless steel sidewalls. Stimuli of various voltages (0–2 V) are applied for 10 ms every 30 min over the 24-h period. Video clips including 30 s of data prior to and after the stimulation are automatically generated (see Video S2). Object velocity (lower panels) and shape change (data not shown) are used to score response, together with visual inspection of the video clips. In the example shown, a stimulus of 1 V for 10 ms is applied at time “0” in two fish. Behavior is scored as “sleep” or “active” based on the existence of movements prior to the stimulation and the video clip. Responses are classified as absent (top) or present (bottom). Note response in fish with higher baseline activity (bottom). (E) Electrical stimulus dose response experiments in fish in active or sleep states. Pulses randomly ranging from 0 to 2 V were applied every 30 min through the day and night. Response and sleep/active status were scored, with no habituation noted. The results presented here used a definition of 6 s of prior inactivity as “sleep.” The curve depicts the percentage of stimuli eliciting responses (movement) at increasing voltage strengths in fish demonstrating prior sleep or active states. Numbers indicate the number of stimulus instances and number of animals (in parentheses). Note that as voltage increased, progressively more animals demonstrated a response, but animals scored as active reacted to lower voltages (*p <* 0.01 at voltage ranging from 0.25 to 0.5 V using Chi^2^). At high voltage, all animals demonstrated a response regardless of prior activity state (i.e., ∼100% at 2 V). Stimuli of 0.38 V (arrow) produced the largest differential response between fish in active and sleep states (bar graph). (F) Quantitative ROC analyses were performed to examine the optimum interval of prior inactivity (<6 pixels/s) that was associated with increased arousal threshold [56]. SE and SP for response to stimulation were computed for animals in active and inactive states. Note that 6 s of prior inactivity was the best discriminating value in terms of SE and SP, and was thus adopted to form our definition of sleep in adult zebrafish (boxed area indicated by arrow). (G) Place preference and characteristic posture during sleep. Left panels show characteristic posture (drooping caudal fin) during sleep (for typical example, see also Video S1). Right panel shows location of sleep occurrences (<6 pixels/s for at least 6 s) plotted as dots over the 24-h period. Sleep occurred either near the surface of the water or at the bottom of the recording chamber, with some interindividual preferences. (H) Sleep bout length (in seconds) in a single fish, integrated every 10 min over 2 d. For every 10 min, the mean length of inactivity periods lasting at least 6 s is calculated. Note presence of some sleep bouts even during the day. (I) Mean percent sleep time of 13 normal adult fish (± standard error of the mean, represented as shaded areas) across the light/dark cycle, after a 3-d habituation period. Data are plotted as the mean of 2-h blocks. Note the extremely strong nocturnal preference for sleep in adult zebrafish (5.1% ± 1.3% of time during the day, 58.1% ± 3.3% of time during the night).

We first performed a fine analysis of the characteristics and architecture of the sleep-like state in fully formed adults to develop a working definition of sleep in the zebrafish. We used videotracking of fish illuminated by an infrared source under light and dark conditions (adult fish sleep recording system [AFSRS]; Figure 1A; see also Materials and Methods), and found that zebrafish adults are strongly diurnal, displaying higher activity during the day (Figure 1B and 1C). Brief periods of inactivity, often associated with a drooping caudal fin, were observed, suggesting a sleep-like state (Video S1). These periods of inactivity alternated with active periods during both the night and the day (Figure 1B). Further, the state was easily reversible by gentle tapping, acoustic stimulation, or weak electrical field stimulation (e.g., 2–6 V).

One important characteristic of sleep is increased arousal threshold. To study this phenomenon, the reaction of fish to a weak electrical field of variable strength was studied (Video S2). Random voltage stimuli (0–2 V) were applied every 30 min through the day and night (Figure 1D). We noted that fish in an active state were more likely to respond to very low voltage stimuli than those that were inactive (see legend to Figure 1 for details). At higher voltages, all fish responded regardless of activity state (Figure 1E). Inactivity was thus associated with an increased arousal threshold to electrical stimulation, with the greatest differential response observed at 0.38 V. Sub-analyses performed during the day versus the night, and in individual animals, yielded similar results (data not shown).

We next defined the minimum epoch of immobility distinguishing sleep from simple inactivity. To do so, we used receiver operator curve (ROC) analysis [56] of the results of the electrical stimulation experiments. In this analysis, sensitivity (SE) and specificity (SP) (and Kappas) for response to stimulation in inactive versus active states are plotted as the discrimination voltage threshold is varied. A true positive is defined as a demonstration of sleep, as defined by immobility and non-response to stimulation. SE is defined as the percent of non-response to stimulation when inactive/total number of trials when inactive. SP is defined as the percent of response when active/total number of trials when active. These analyses considered the percentage of responses to differing voltages (0.13, 0.38, 0.5, 1 and 2 V) as well as the consecutive number of seconds (0–30 s) of inactivity (<6 pixels/s) preceding the stimulus. These SE/SP pairs are plotted on ROC and qualitative ROC planes. Diagnostic lines are drawn on the qualitative ROC plane, and the ideal test point is plotted. This analysis indicated that the best SE/SP ratio (point closest to ideal test point and closest to the diagnostic line) was obtained when using 0.38 V and 6 s of prior inactivity. As expected, SE increased with increasingly longer periods of inactivity, as the longer the prior period of inactivity was, the likelier true sleep (without reactivity to electrical stimulation) was observed (Figure 1F). In contrast, SP decreased with increasingly longer periods of inactivity, as more and more short periods of true sleep were missed and considered “active.” This analysis provided a working definition of zebrafish sleep: an interval of inactivity (<6 pixels/s) lasting at least 6 s (Figure 1E and 1F). All other periods were defined as active (awake). Using this definition, we determined that most sleep episodes occurred at the bottom or the top of the tanks (Figure 1G) and were remarkably consolidated during the night (Figure 1H and 1I). No sex differences were found in sleep amounts or distribution (data not shown).

### Sleep Deprivation and Recovery in the Dark

**Figure 2:**
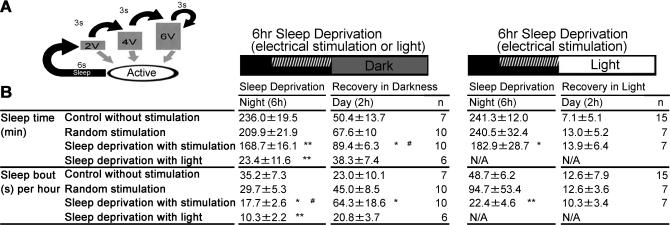
Homeostatic Regulation of Zebrafish Sleep (A) Illustration of the sleep deprivation protocol. An electrical pulse (2 V, 10 ms) is applied when the target fish displays sleep. When the fish does not respond within 3 s, the stimulus is increased stepwise (2 V per step) to a maximum of 6 V. If the fish does not react to the 6-V stimulus, stimulation is repeated. A yoked control fish is stimulated at the same time in a different chamber (independent of its sleep/wake state), to control for the stress of the procedure. For video recordings of sleep-deprived fish at the beginning of the stimulation period and at the end of the 6-h procedure, see Videos S3 and S4, respectively. (B) Quantitative effects of sleep deprivation using electrical stimulation or light, as applied 6 h prior to usual light onset, with release in the dark or the light. The procedure induces a ∼30% loss of sleep over the 6-h period, while yoked stimulated controls have ∼10% less sleep. Groups are compared using ANOVA with grouping factors (all models significant, except recovery in light), followed by post hoc testing and Bonferroni corrections. A greater amount of recovery sleep is observed in sleep-deprived animals than in undisturbed controls and yoked stimulated animals (#, *p <* 0.05 sleep-deprived versus controls; *, *p <* 0.05 sleep-deprived versus yoke controls; **, *p <* 0.01). A partial rebound is also observed in yoked stimulated controls. Sleep bout length also increased during the rebound period. Interestingly, light almost completely suppressed sleep (∼90%) without any apparent recovery (even when monitoring was conducted for a subsequent 48 h; data not shown). Similarly, sleep deprivation by electrical stimulation is not associated with a sleep rebound when animals are released in the light (right panel).

We next investigated whether sleep episodes are homeostatically regulated by observing sleep-deprived animals. To do this, we first attempted sleep deprivation by tapping on the aquarium walls or using noise introduced through an underwater speaker. As we noted rapid habituation, electrical stimulation was next attempted. Although this procedure was also imperfect, as we found it extremely difficult to keep the fish awake in the dark for long periods of time (Video S4), it was retained as the method of choice, as it did not result in rapid habituation. We next designed a computerized system to electrically stimulate a fish each time it displayed sleep behavior (Figure 2A). A yoked control fish was stimulated concurrently at the same voltage (though not necessarily while resting) in a separate chamber to control for stress. Increased voltage from 2 to 6 V was applied to both fish if the inactive fish did not react to stimulations (Figure 2A).

Animals were sleep deprived during the 6 h of the dark prior to usual light onset (9 a.m.), and released into either the usual light (150 lux) or an extended period of darkness. This procedure successfully induced sleep deprivation, although partial habituation was observed after 4 h of stimulation, i.e., toward the end of the procedure (data not shown). Indeed, sleep-deprived animals appeared increasingly immobile and unreactive to stimulation toward the end of the procedure, in contrast to yoked control stimulated fish (Videos S3 and S4). Further, as sleep episodes normally occur at high frequency at night, random stimulation in the control fish also induced mild partial sleep deprivation (while controlling for stress). Overall, we found that this procedure induced a 30% decrease in sleep in the sleep-deprived group versus undisturbed controls. In contrast, a 10% decrease in sleep was observed in the yoked control group, representing a milder degree of sleep deprivation (Figure 2B) and providing a dose-response curve of increasing amounts of sleep deprivation.

After release into an extended period of darkness during the subjective day, sleep in undisturbed control animals was lowest. In yoked control animals (partially deprived), minor recovery sleep was observed, while a significant rebound was observed in the sleep-deprived animals, indicating homeostatic regulation of sleep. Differences were statistically significant between sleep-deprived versus yoke control stimulated or undisturbed fish (Figure 2B). Sleep bout length was also increased in both the yoke control and sleep-deprived groups, although not significantly in the yoke control, compared to the undisturbed group.

### Light Suppresses Sleep and Produces No Rebound

Remarkably, a sleep rebound was not observed when sleep-deprived animals were released into light (Figure 2B). Further, when fish were exposed to 150-lux light during the last 6 h of the biological night, but not electrically stimulated, there was a dramatic suppression of sleep (90% decrease) that was not followed by a rebound when animals were released into darkness (Figure 2B). A similar, nearly complete suppression of sleep was also observed when animals were kept under constant light conditions for 3 d (Table 1). Again, no significant rebound was observed during the day or the following nights (data not shown). During longer exposure to constant 150-lux light, a progressive return of sleep was noted over a period of 1–2 wk (Figure S1). As previously reported for activity [57], sleep was modulated by circadian influences under constant light and dark conditions (Table 1). Unlike most mammals, however, the direct effect of dark and, more strikingly, light was stronger than circadian influences. Indeed, for most parameters, values varied significantly more with light exposure than with circadian timing (Table 1).

**Table 1:**
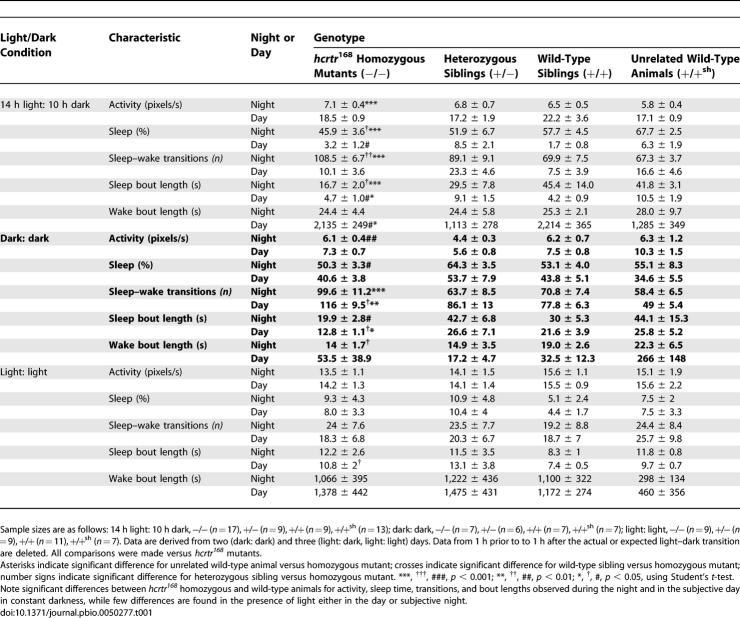
Activity and Sleep Characteristics in *hcrtr* ^168^ Homozygous Fish, when Compared to Heterozygous Siblings, Wild-Type Siblings, and Unrelated Wild-Type Animals in Various Light/Dark Conditions

### *hcrtr* Is Widely Expressed in the Brain of Zebrafish Embryos

**Figure 3:**
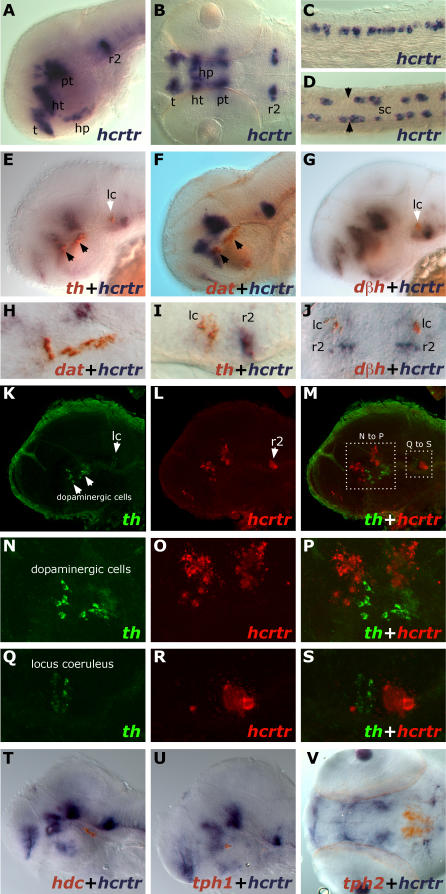
*hcrtr* Is Not Expressed in Monoaminergic Nuclei at 2 dpf Results were all obtained using ISH. (A–D) *hcrtr* is expressed in the brain and spinal cord. (A) Lateral view of *hcrtr* expression in telencephalon (t), hypothalamus (ht), hypophysis (hp), posterior tuberculum (pt), and ventral rhombomere 2 (r2). (B) Dorsal view of *hcrtr* expression in the same structures. (C) Lateral view of spinal cord *hcrtr* expression. (D) Dorsal view of spinal cord (sc) *hcrtr* expression. Note the expression at the periphery of the spinal cord. Limits between somatic muscles and spinal cord tissue are indicated by black arrowheads. (E–J) Two-color ISH, flat mounts. (E) Lateral view of a hemi-brain stained for *hcrtr* (blue) and *tyrosine hydroxylase* (*th*, red); locus coeruleus (lc) is indicated by a white arrowhead; dopaminergic clusters by black arrowheads. (F) Lateral view of a hemi-brain stained for *hcrtr* (blue) and *dopamine transporter* (*dat*, red). (G) Lateral view of hemi-brain stained for *hcrtr* (blue) and *dopamine beta hydroxylase* (*dbh*, red). (H) Lateral close-up showing the absence of *hcrtr* expression in dopaminergic cells. (I) Lateral close-up showing absence of *hcrtr* expression in the locus coeruleus. (J) Dorsal close-up confirming absence of colocalization in the locus coeruleus. (K–S) Double fluorescent ISH, confocal microscopy pictures (stacks of 0.5- or 1-μm sections). (K and L) Lateral view of a hemi-brain stained for *tyrosine hydroxylase* (K) (*th*, green) and *hcrtr* (L) (red). (M) Merged view; note absence of yellow, indicating no colocalization. (N–P) From boxed area in (M), close-up of the diencephalic dopaminergic region and absence of *hcrtr* colocalization (P). (Q–S) From boxed area in (M), close-up on the locus coeruleus region and absence of *hcrtr* colocalization (S). (T) Lateral view of a flat-mounted hemi-brain stained for *hcrtr* (blue) and *histidine decarboxylase* (*hdc*, red). Note absence of colocalization. (U and V) Flat mounts. Lateral (hemi-brain) and ventral views of embryos stained for *hcrtr* (blue) and *tryptophan hydroxylase 1 (tph1)* (U) and *tph2* (V), respectively. Note absence of colocalization.

To investigate functional conservation of neurotransmitters regulating sleep in zebrafish, we next anatomically and functionally studied the hypocretin system, the only system known to cause a primary sleep disorder (narcolepsy) in mammals. In conjunction with previous work on the hypocretin neuropeptide [48], we identified a single hypocretin receptor in *Tetraodon* and in zebrafish (*hcrtr* [also known as *hcrtr2*]) through bacterial artificial chromosome (BAC) library screening and *in silico* database searches (see Materials and Methods). As recently noted [50], zebrafish Hcrtr has higher homology to mammalian HCRTR2, the subtype of primary importance in the mediation of the narcolepsy phenotype [12]. As in mammals [19], we found widespread *hcrtr* expression in the telencephalon, hypothalamus, hypophysis, posterior tuberculum, and hindbrain (Figure 3A and 3B) and in selected spinal cord neurons (Figure 3C and 3D) of larvae at age 2 d postfertilization (dpf). Limited expression was found in thalamic and pallidal areas, reminiscent of overall mammalian HCRTR1 and HCRTR2 mRNA distribution (cortex and hippocampus, basal forebrain, central midline thalamic areas, hypothalamus, and brainstem) [19], although overall neuroanatomical correspondence of structures between these species is only partially established [41].

### *hcrtr* Is Not Localized on Major Monoaminergic Cell Groups in Zebrafish Embryos

We next simultaneously mapped the distribution of *hcrtr* with that of monoaminergic cell groups. In mammals, monoaminergic cell groups modulate wakefulness and are among the most hypocretin-receptor-rich brain regions [19]. These are stimulated by hypocretins and are commonly assumed to mediate much of the downstream effects of hypocretin on sleep regulation [12,58]. Interestingly, however, using double in situ hybridization (ISH) on 2-dpf larvae, we saw no significant colocalization with adrenergic (Figure 3G and 3J), dopaminergic (Figure 3E–3S), histaminergic (Figure 3T), or serotoninergic (Figure 3U and 3V) neurons. Flat mounts and close-ups confirmed these results (Figure 3H–3J). Double fluorescence ISH followed by confocal microscopy (Figure 3K–3S) also demonstrated an absence of colocalization in dopaminergic and adrenergic cells, in contrast to a previous report [50].

### *hcrtr* Expression in Adult Zebrafish Brain

**Figure 4:**
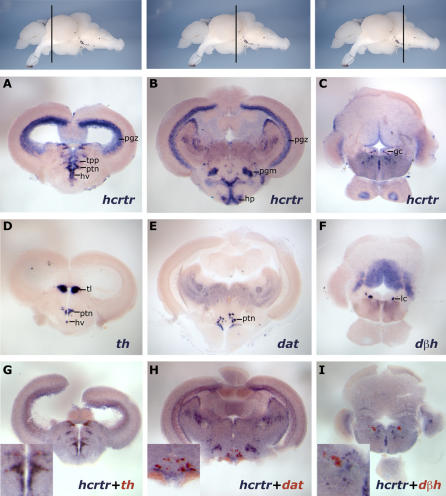
*hcrtr* Is Expressed in Neither the Posterior Diencephalic Dopaminergic Cells nor the Locus Coeruleus of Adult Zebrafish Brain The topmost panels display lateral views of zebrafish adult brains with transversal plane corresponding to sections presented below. Results were all obtained using ISH. (A–C) *hcrtr* mRNA is expressed in the periventricular gray zone of optic tectum (pgz), periventricular nucleus of the posterior tuberculum (tpp), posterior tuberal nucleus (ptn), and ventral zone of periventricular hypothalamus (hv) of the anterior diencephalon (A). In the posterior diencephalon (B), *hcrtr* mRNA is mainly detected in the periventricular gray zone of optic tectum, medial preglomerular nucleus (pgm), and hypophysis (hp). *hcrtr* has a sparse expression in the anterior rhombencephalon (C), including the griseum centrale (gc) near the locus coeruleus. (D–F) *th, dat,* and *dbh* expression patterns in sections similar to those shown in (A–C), respectively. *th* is expressed in posterior tuberal nucleus and ventral zone of periventricular hypothalamus and in the torus longitudinalis (tl) (D). *dat* expression is restricted to a few posterior tuberal nucleus cells in the posterior diencephalon (E). *dbh* is expressed in the locus coeruleus (lc) (F). (G–I) Double ISH on equivalent sections showing colocalization of *hcrtr* (blue) with *th* (red) in the anterior catecholaminergic/dopaminergic cells of the diencephalon (G), absence of colocalization of *hcrtr* (blue) with *dat* (red) in the posterior catecholaminergic/dopaminergic cells of the diencephalon (H), and absence of colocalization of *hcrtr* (blue) with *dbh* (red) in the catecholaminergic/noradrenergic locus coeruleus (I).

To determine whether connectivity of the hypocretin and monoaminergic systems emerges later in development, we also performed ISH on adult zebrafish brain sections, using the adult zebrafish atlas established by Wulliman and colleagues [42]. The embryonic distribution of *hcrtr* was broadly maintained in the adult brain, with prominent localization in the telencephalon, hypothalamus, posterior tuberculum, hypophysis, and brainstem cranial nuclei. In addition to the areas of detected expression in 2-dpf embryos, notable expression was also observed in the periventricular gray zone of the optic tectum (Figure 4A–4C). As in embryos, colocalization of *hcrtr* with monoaminergic cells was absent except for a few anterior dopaminergic neurons (Figure 4G). Most notably, and unlike previously reported [50], expression was absent in the large majority of diencephalic dopaminergic neurons (Figure 4B, 4E, and 4H) and in the locus coeruleus (Figure 4C, 4F, and 4I). In this last area, however, a few receptor-positive cells were present immediately medially to the locus coeruleus (Figure 4I). Labeling of the locus coeruleus was performed using *dbh* (Figure 4I), *th,* and *adra2a* (data not shown).

### Non-Monoaminergic Locations of *hcrtr* Expression

**Figure 5:**
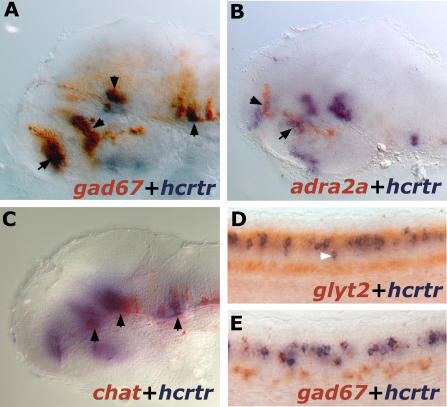
*hcrtr* Coexpression with GABAergic, Adrenergic, Cholinergic, and Glycinergic Systems in 2-dpf Larvae Results were all obtained using ISH. (A) Lateral view of a flat-mounted hemi-brain stained for *hcrtr* (blue) and *gad67* (red). Note multiple areas of overlap indicated by black arrowheads. (B) Lateral view of a flat-mounted hemi-brain stained for *hcrtr* (blue) and *adra2a* (red). Double staining in the anterior hypothalamus and telencephalon is indicated by arrowheads. (C) Lateral view of a flat-mounted hemi-brain stained for *hcrtr* (blue) and *chat* (red). Double-stained regions are indicated by black arrowheads. (D) Lateral views of spinal cord stained for *hcrtr* (blue) and *glyt2* (red). Note that most *hcrtr* neurons but not all (white arrowhead) are glycinergic. (E) Lateral views of spinal cord stained for *hcrtr* (blue) and *gad67* (red). Note that all *hcrtr* neurons are GABAergic.

As *hcrtr* is not significantly colocalized with major monoaminergic neurons in embryos, we next surveyed zebrafish embryos with other neuronal markers (acetylcholine, GABA, glycine, and glutamate markers) that have been proposed as hypocretin targets in various sleep regulatory models [2,58]. We found that Adra2a, Gad67 (also known as Gad1), ChAT, and Glyt2 (also known as Slc6a5) were expressed in regions similar to where *hcrtr* was expressed (Figure 5). Double ISH further confirmed that most *hcrtr*-positive cells were Gad67-positive GABAergic cells (Figure 5A), except in the hypophysis and the ventral posterior tuberculum. A subpopulation of *hcrtr*-positive GABAergic cells in the anterior hypothalamic area was also positive for Adra2a (Figure 5B). Some overlap was also observed between the cholinergic system and *hcrtr*-positive cells in the diencephalon and in rhombomere 2 (Figure 5C). In the spinal cord, *hcrtr-*positive neurons were neither primary sensory neurons nor motoneurons, but were located closer to the primary sensory neuron layer, a region that could be equivalent to lamina-II in mammals; this area is involved in the secondary processing of sensory information such as pain [59]. Most of these neurons were glycinergic (Figure 5D) and GABAergic (Figure 5E). In mammals, equivalent neurons receive dense hypocretin projections and are stimulated by the peptide, with a role in the modulation of nociceptive input [59–62].

**Figure 6:**
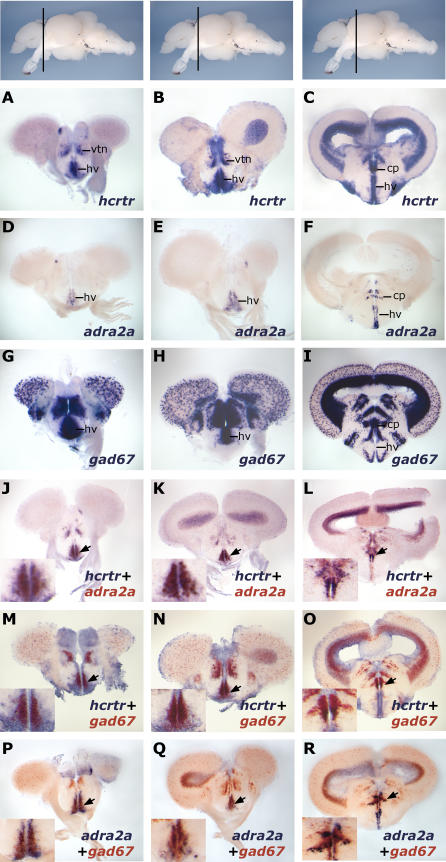
A Population of Anterior Hypothalamic *hcrtr-*Positive Neurons is *adra2a-* and *gad67*-Positive The topmost panels display lateral views of zebrafish adult brain with transversal plane corresponding to sections presented below. Results were all obtained using ISH. (A–I) Single ISH expression patterns of *hcrtr* (A–C), *adra2a* (D–F), and *gad67* (G–I) in the anterior diencephalon. In the most rostral part of the diencephalon, *hcrtr* is mainly expressed in the ventral thalamic nucleus (vtn) and the ventral zone of the periventricular hypothalamus (hv) (A and B). Posteriorly, it is mostly expressed in the peripheral gray zone and in periventricular areas including the central posterior thalamic nucleus (cp) and the ventral zone of the periventricular hypothalamus. (J–L) Double ISH showing colocalization of *hcrtr* and *adra2a* in the ventral zone of the periventricular hypothalamus (arrowheads). (M–O) Double ISH showing colocalization of *hcrtr* and *gad67* in the ventral zone of the periventricular hypothalamus and ventral thalamic nucleus (arrowheads). (P–R) Double ISH showing colocalization of *gad67* and *adra2a* in the ventral zone of the periventricular hypothalamus (arrowheads). Note that *hcrtr, gad67,* and *adra2a* only colocalize in the anterior hypothalamus and not in the posterior ventral zone of the periventricular hypothalamus, where *gad67* is absent (I). Posterior to this area, only the central thalamic nucleus expresses these three markers (last column).

**Figure 7:**
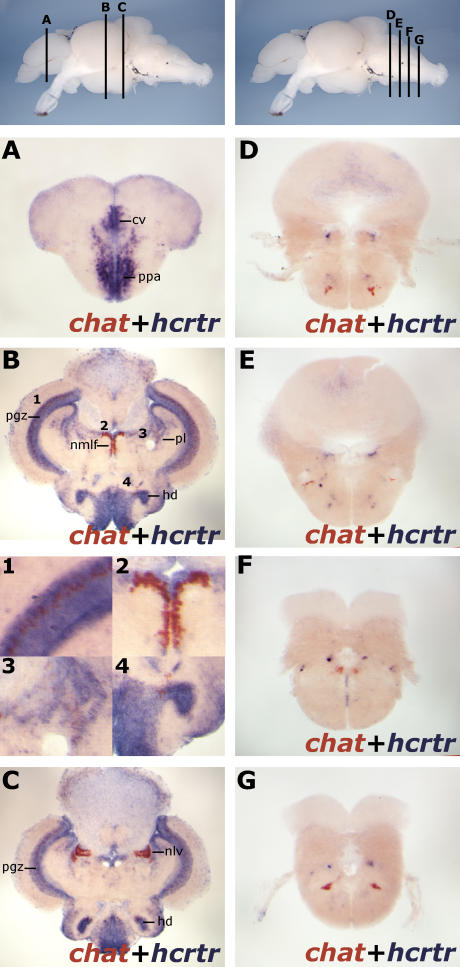
*hcrtr* Does Not Colocalize with the Cholinergic System in the Telencephalon and Rhombencephalon The topmost panels display lateral views of zebrafish adult brains with transversal planes corresponding to sections presented below. Results were all obtained using ISH with *chat* and *hcrtr*. (A) Cholinergic neurons were not detected in the adult zebrafish telencephalon using *chat* or *vacht* (data not shown). In this area, *hcrtr* is strongly expressed in the commissural nucleus of the ventral telencephalic area (cv) and the anterior part of the parvocellular preoptic nucleus (ppa). (B and C) Major clusters of cholinergic neurons are present in the periventricular gray zone (pgz) ([B], panel 1), the nucleus of the medial longitudinal fascicle (nmlf) ([B], panel 2), the perilemniscal nucleus (pl) ([B], panel 3), the dorsal zone of periventricular hypothalamus (hd) ([B], panel 4), and the nucleus lateralis valvulae (nlv) (C). Coexpression with *hcrtr* was mostly observed in the periventricular gray zone ([B], panel 1) and in the dorsal zone of periventricular hypothalamus ([B], panel 4). (D–G) A careful survey of the rhombencephalon failed to reveal coexpression of *hcrtr* with *chat*-positive neurons, including in the peri-locus coeruleus area.

In a second analysis, double ISH data extended in adults, with a primary focus on the hypothalamic area. As in embryos, we found that many *hcrtr*-positive cells were Gad67-positive. In the anterior hypothalamus and ventral thalamic nucleus (Figure 6, first two columns), most *hcrtr*-positive cells were GABAergic, starting at the diencephalic–telencephalic junction. Further, the majority of the anterior hypothalamic GABAergic cluster was Adra2a-positive. In the posterior diencephalon, only a small region of the central posterior thalamic nucleus was Adra2a- and Gad67-positive (Figure 6, last column). Studies using cholinergic markers were next performed (Figure 7). Our primary focus was on the telencephalon and the pons, where cholinergic cells equivalent to sleep regulatory neurons of the nucleus basalis and laterodorsal tegmentum/pedunculopontine nuclei have been reported. Cholinergic staining was abundant in the diencephalon and rhombencephalon, including in many cranial nerve nuclei. Colocalization with *hcrtr* was only observed in a few areas, most notably in the peripheral gray zone of the optic tectum and periventricular hypothalamus (Figure 7B and 7C). In the telencephalon, we failed to detect any cholinergic neurons (Figure 7A); this population has been found only in some fishes. Similarly, close to the locus coeruleus, where the equivalent of the laterodorsal tegmentum/pedunculopontine cells are believed to be located, no ChAT expression was noted (Figure 7D–7G).

### Behavioral Characterization of a *hcrtr* Null Mutant

**Figure 8:**
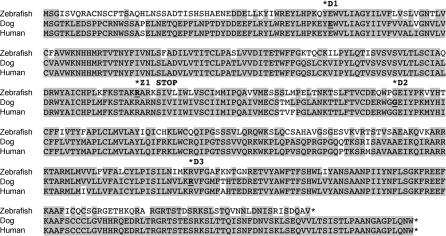
Known Null Mutations of Zebrafish and Mammalian *hcrtr* Genes Note the extremely high homology with mammalian HCRTR2 (71% identity with human HCRTR2). Z1: an HCRTR arginine to stop (“STOP”) was identified through TILLING. D1: an E54K substitution null allele previously identified in dog (dachshund). D2 and D3: positions of HCRTR2 frame shifts in dog (doberman, D2, and labrador, D3) were followed by 32 and two amino acids, respectively, before truncation.

We next screened an ethylnitrosurea-mutagenized TILLING (for “targeting induced local lesions in genomes”) library for *hcrtr* mutations. A premature stop codon mutation (R168 to stop) was identified *(hcrtr^168^)* that results in the predicted loss of four transmembrane domains as well as the intracellular loop 3 domain required for G-protein coupling. The truncation is also located upstream of two mutations known to produce an inactive protein resulting in canine narcolepsy [12] (Figure 8). Homozygous *hcrtr^168^* animals developed normally into viable and fertile adults. Extensive observation of larvae and adults did not yield any obvious phenotype, such as the occurrence of sudden REM-sleep-like paralysis episodes (e.g., cataplexy or cataplexy-like behaviors) characteristic of mammalian narcolepsy [12,14,31,63], either spontaneously or when excited by food administration or mating. Similarly, activity monitoring did not reveal any large differences between mutant, heterozygous, and wild-type larvae of similar background (data not shown).

**Figure 9:**
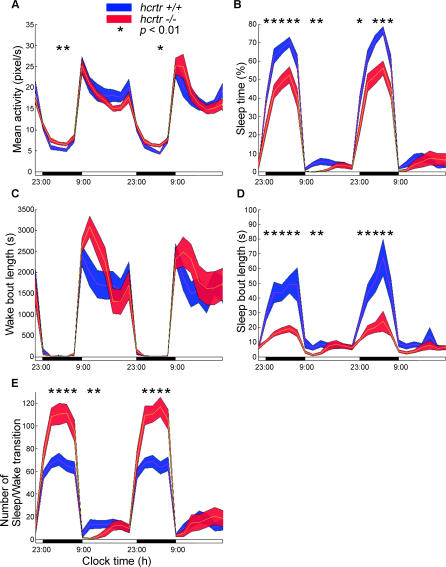
Sleep–Wake Patterns in *hcrtr^168^* Mutants versus Wild-Type Recordings were performed using the AFSRS over 48 h after 3 d of habituation. Data are plotted every 2 h. (Wild-type, *n =* 22; homozygous mutant, *n =* 17). (A) Activity patterns. Note slightly increased activity (+20%) in mutant fish during the night. (B) Percent sleep per hour. Note decreased sleep amounts (−30%) in mutant fish during the entire night. (C) Mean wake bout length per hour (calculated as the mean period length of the non-sleep periods each hour) is similar in both genotypes. (D) Mean sleep bout length per hour. Note dramatic decrease in sleep bout length (−65%) during the night in mutant animals. (E) The number of sleep–wake transitions per hour increases dramatically in *hcrtr^168^* mutants during the night (+60%). For overall means of these parameters across the dark and light periods, and during other light cycles, see Table 1.

We next studied adult wild-type and mutant fish using our AFSRS (including comparison of heterozygous and wild-type siblings within the same family) under typical light/dark conditions. We found that activity of *hcrtr^168^* mutants was slightly increased (Figure 9A; Table 1) and sleep amounts were decreased by 20%–30% during the night (Figure 9B; Table 1). Most strikingly, fine architecture analysis revealed a 60%–70% increase in the number of sleep–wake transitions, and a 60% decrease in sleep bout length during the night, indicating sleep fragmentation in *hcrtr^168^* (Figure 9D and 9E). Heterozygous animals generally behaved as wild-type siblings, although in some measures (e.g., sleep time and sleep transitions), an intermediary phenotype was observed (Table 1). Activity and sleep architecture were normal during the day in all genotypes. Further, wake bout length was essentially unchanged during the day or the night (Figure 9C; Table 1). These data indicate that the hypocretin receptor is required for proper sleep regulation in adult zebrafish under light/dark conditions.

Studies under constant light and dark indicated significantly decreased sleep amounts and significant sleep fragmentation in *hcrtr^168^* compared to wild-type animals at all circadian time points, but these effects were masked by the stimulating effect of light (Table 1). Similar increases in locomotion (and decreased sleep) were observed in all three genotypes when animals were newly moved from their usual aquaria to the recording chambers; thus, disruption of nocturnal sleep in the mutant was unlikely to be due to differential effects of stress or the food deprivation associated with our monitoring method (Figure S2). Further, food intake satiety monitoring studies (Figure S3) and studies of locomotor activation after feeding were performed, and all genotypes reacted similarly (Figure S4).

### Effects of Icv Injections of Hypocretin-1 or Hypocretin-2 in Wild-Type Animals and *hcrtr^168^* Mutants

**Figure 10:**
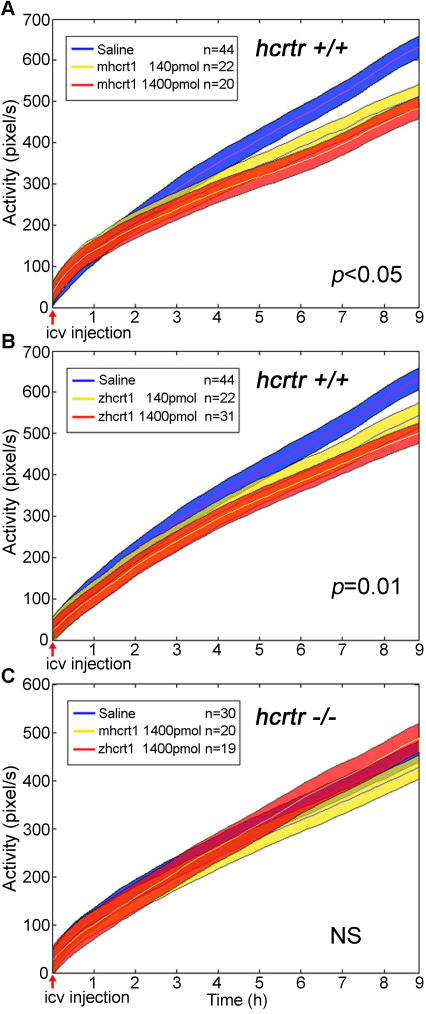
Effects of icv Injections of Hypocretin-1 on Activity in Wild-Type Zebrafish versus *hcrtr^168^* Mutants Injections were performed as described in Materials and Methods, 5–10 min prior to recordings. (A) Cumulative activity in wild-type fish over 9 h in the recording chamber after icv injection of saline (blue) or 140 (yellow) or 1,400 (red) pmol of mammalian hypocretin-1. The initial steep rise in activity is due to habituation to the novel environment. Note long-lasting, dose-dependent reduction of activity after 140- and 1,400-pmol injections. A dose-dependent significant decrease in locomotion was detected using multilinear analysis of variance with repeated measures with increasing hypocretin doses (*p <* 0.05) as grouping factors. (B) Cumulative activity in wild-type fish over 9 h after icv injection of saline (blue) or 140 (yellow) or 1,400 (red) pmol of zebrafish hypocretin-1. Note dose-dependent reduction of activity after 140- and 1,400-pmol injections, as observed after mammalian hypocretin-1. A dose-dependent significant decrease in locomotion was detected using multilinear analysis of variance with repeated measures with increasing hypocretin doses (*p* = 0.01) as grouping factors. (C) Cumulative activity in *hcrtr^168^* mutants over 9 h after icv injection of saline (blue) or 1,400 pmol of mammalian (yellow) or zebrafish (red) hypocretin-1. Note absence of sedative effects after zebrafish or mammalian hypocretin-1 in *hcrtr* mutants, when compared to saline. *n,* number of animals tested at each dose.

Hypocretin-1 icv injections are wake-promoting and increase locomotion in mice, rats, and dogs [27,28,30]. In contrast, hypocretin-2 is generally inactive because of rapid catabolism [64]. Prior to usual light–dark transition time, adult zebrafish were briefly anesthetized, and hypocretin peptides (or saline) were injected icv. Animals were subsequently released in the dark while activity and sleep were measured using the AFSRS. In controls, locomotion was high (novel environment), followed by habituation and reduced activity/increased sleep in the dark (Figure 10A and 10B). In hypocretin-1-injected fish, a reduction in locomotor activity was observed (Figure 10A and 10B). This effect was dose dependent and occurred with either the human or zebrafish hypocretin-1 peptide. Based on sleep scoring of these data, we found that both mammalian and zebrafish hypocretin-1 significantly increased total sleep time (23.0% ± 11.3% and 28.7% ± 9.0% above baseline sleep, respectively, *p <* 0.05 in both cases after 1,400 pmol) during 9 h of continuous recording (Figure 10). As expected, zebrafish hypocretin-2 was inactive in wild-type zebrafish (data not shown). In the TILLING *hcrtr^168^* mutant, the sleep-promoting effects of mammalian or zebrafish hypocretin-1 were abolished (Figure 10C). Overall, these experiments indicate antagonism of the sleep-promoting effects of icv hypocretin-1 injection by *hcrtr^168^*, confirming that the effect is mediated through Hcrtr.

## Discussion

Our experiments demonstrate that rest episodes in adult zebrafish represent a genuine sleep-like state, characterized by reversible periods of immobility, place preference (bottom or surface), circadian regulation, and homeostatic rebound. Interestingly, unlike in larvae [50,51,54], sleep deprivation was difficult to achieve in adults and was associated with a sleep rebound that was only detectable when the fish were released in the dark. Our study is unique as we studied adults and demonstrated that only 6 s of prior inactivity was sufficient to be associated with decreased arousal threshold and thus to qualify as sleep. Other studies have been primarily performed in larval zebrafish and did not test or report on intervals shorter than 1 min [50,51]. In agreement with our finding, a preliminary report in adults reported that long-term sleep deprivation using a moving partition technique or electric shock produced a sleep rebound, associated with increased arousal threshold [54].

Unlike in most mammals, we also found that even moderate levels of light exposure have strong sleep-suppressant effects in zebrafish, and that circadian regulation has a more minor role. The light suppressant effect was not associated with deleterious behavioral effects over a week, but sleep reappeared progressively after 8 d (Figure S1). These results are in agreement with data from Hurd and colleagues [57], who found that only a portion of adult fish displayed detectable circadian activity rhythms under constant light or dark conditions at 28 °C, in all cases with significantly lower amplitude than under alternating light/dark conditions. Most strikingly, we also found that light was not only able to suppress sleep (Figures 2 and S1) but that no sleep rebound was observed upon release in the dark. In goldfish and perch, a sleep rebound in the light has been found after sleep deprivation by light, but was mild [7]. Further, the lack of rebound after sleep deprivation by light in our experiment contrasts with the observation of homeostatic regulation after a much lower level of sleep deprivation using electrical stimulation. Overall, whereas it is likely that homeostatic regulation of rest can be demonstrated in some circumstances (in our case, electrical stimulation when sleeping in the dark), we found that wake induced by light in zebrafish was not, on a short-term basis, associated with a mounting sleep debt.

How could this unusual effect of light be explained? Unlike in mammals, but as reported in *Drosophila,* most zebrafish cells are directly photoreceptive [65,66]. Further, melatonin is a strong hypnotic in this species, a property that may be related to the diurnal pattern of activity of zebrafish [51]. The combined effect of light on various cell populations, together with its suppressive effects on melatonin production, may result in multiple redundant wake-promoting inputs into the brain. In favor of this hypothesis, variable effects of light have been observed in other teleosts, where it acts to suppress rest and induce rest rebound in the perch and goldfish [7], both diurnal fish, whereas it has calming effects in nocturnal fish such as the tench [6]. In this context, the strong effects of light or melatonin may be able to overcome the more minor regulatory effects of other neural networks regulating sleep homeostasis in zebrafish.

Recent results in other species, most notably in diurnal birds, indicate that some vertebrates have sleep regulatory characteristics similar to those of zebrafish. Birds are especially interesting as, unlike fish, it is possible to document all the electroencephalographic characteristics of mammalian sleep [6]. Migratory sparrows, for example, are able to survive for long periods of time without sleep under selected ecological conditions and are extremely sensitive to light and dark [67]. Similarly, sleep in pigeons is strongly suppressed by light, without electroencephalogram-defined non-REM sleep rebound in darkness [68–70]. Like zebrafish, diurnal birds such as pigeons are also remarkably sensitive to melatonin, and do not exhibit wake rebound after melatonin-induced sleep [71]. It may thus be that the need for homeostatic regulation of sleep has not strongly evolved in zebrafish, and that it is not as universal in vertebrates as previously believed. Rather, in both diurnal birds and fish, the direct effect of light or melatonin may be able to bypass homeostatic regulation of sleep. Further studies of sleep deprivation by light versus other methods in these species may reveal molecular mechanisms regulating sleep homeostasis.

Our studies have also shown significant and informative divergence in the organization of the hypocretin system in zebrafish. We have described a small group of approximately 20 hypocretin cells in the preoptic hypothalamus of embryonic zebrafish and characterized a compact promoter driving expression in these cells [48]. ISH and immunochemistry in adult brains indicates that approximately 40 cells are present in the adult zebrafish preoptic area (data not shown and Kaslin et al. [49]), although an additional more anterior group, probably detected through antibody cross-reactivity, was found using immunochemistry with mammalian antibodies [48,49]. The *hcrt* cluster in zebrafish is distal to the histaminergic cell group expressing histidine decarboxylase, unlike in mammals where these two cells groups are closely adjacent within the posterior hypothalamus.

Unlike mammals, the zebrafish has only one hypocretin receptor. This result, surprising when considering the frequency of gene duplications in this species, was confirmed through *in silico* searches, BAC library screening, genomic Southern blot analysis, and comparisons of syntenies around HCRTR1 and HCRTR2 in mice, humans, and zebrafish. Indeed, only a single hypocretin receptor is identifiable in current releases of other teleosts (zebrafish, *Fugu, Tetraodon,* medaka, and stickleback). Using ISH, we found that the expression pattern of *hcrtr* is in agreement with overall mammalian hypocretin receptor expression patterns (Figures 3 and 4). Indeed, the high density of hypocretin receptor mRNA in the telencephalon, hypothalamus, posterior tuberculum, and hindbrain, but not lateral thalamic and pallidal areas, is reminiscent of overall mammalian HCRTR1 and HCRTR2 distribution and density (in cortex, hippocampus, basal forebrain, central midline thalamic areas, and hypothalamus) [19], although neuroanatomical correspondence of overall structure across these species is only partially established [41]. Similarly, as in mammals, *hcrtr* is expressed in glycinergic/GABAergic neurons of the spinal cord immediately ventral to sensory neurons. In mammals, these neurons receive dense hypocretin projections and are stimulated by the peptide, with a role in the modulation of nociceptive input [59–62]. Although the overall pattern of expression initially appeared similar to that in mammals, our in-depth analysis indicates differences in expression in relevant sleep regulatory networks (see below).

As in mammals, mutation of the hypocretin receptor disrupts the consolidation of sleep/wake behavior. Unlike narcoleptic humans [14,31], canines [12], and rodents [63], however, *hcrtr^168^* fish do not display sudden episodes of paralysis (cataplexy), which in mammals are believed to represent dissociated REM sleep. Further, *hcrtr^168^* mutant fish do not have decreased wake bout length, whether in the light or the dark. This contrasts with human narcolepsy, where the primary abnormality is an inability to consolidate long periods of wakefulness during the day. Instead, the phenotype of *hcrtr^168^* is only sleep fragmentation and decreased sleep in darkness (e.g., insomnia), another disabling symptom of human narcolepsy [31,32]. Day and night sleep and wake fragmentation, together with episodes of muscle paralysis, are also a primary feature of rodent and canine narcolepsy [12,36,63]. One explanation for this discrepancy is likely to be the strong and direct effect of light in inhibiting sleep in zebrafish during the day, as described above. Indeed, the phenotype of *hcrtr^168^* is not apparent under constant light (Table 1). The direct effect of light in this species may thus have made it unnecessary to evolve a method to consolidate wake, and indeed there was great variability in daytime wake bout length among fish of all genotypes in the absence of light (Table 1).

The lack of wake abnormality in *hcrtr^168^* mutants during the day or the night is also surprising considering recent results indicating increased activity and decreased sleep in zebrafish larvae after heat shock stimulation of hypocretin expression [50]. To address this issue and to test for the specificity of the effects of high doses of hypocretin (as generated in heat shock overexpressing models), we studied the effects of icv injections of mammalian and zebrafish hypocretin-1 at various doses on sleep and locomotion in wild-type zebrafish adults. Mammalian hypocretin-1 is conserved in the functionally important final six C-terminal amino acids of hypocretin-1 and −2 [48,49,72] needed for binding activity, and thus must be active, as reported for *Xenopus* hypocretin [73] and in goldfish experiments. Prober et al. [50] found that heat shock activation of hypocretin expression increased locomotion and wake for several days in larvae, although without altering diurnal fluctuations in activity. In our icv injection experiments, we found that zebrafish or mammalian hypocretin-1, but not zebrafish hypocretin-2, reduces activity and promotes sleep in the dark (Figure 10A). These effects were abolished in the null *hcrtr^168^* mutant, indicating mediation by Hcrtr. The relatively mild reducing effect on locomotion contrasts with the strong locomotor activation and wake-promoting effects of hypocretin-1 reported in mammals. In rats, for example, 8,000 pmol dramatically increases activity [28,30] and sustains wake for 5 h, an effect followed by a proportional sleep rebound lasting 10 h [29].

Overall, although it is difficult to reject the possibility that the increased sleep seen in our experiments after icv hypocretin-1 injection is merely a rebound after a primary wake-promoting effect (an effect that could have been masked by the 10 min of anesthesia and subsequent activation in a novel environment), we believe this hypothesis to be unlikely. Indeed, a very minor increase in locomotion has been reported in goldfish after icv injection of mammalian hypocretin-1 [74], without further follow-up of activity monitoring longer than 1 h after administration. In our experiments, it is difficult to conceive that our injected zebrafish would be activated while in or recovering from anesthesia. Further, even in awake rodents placed in a novel environment, icv hypocretin-1 injection increases locomotion. We thus believe that in zebrafish, unlike in mammals, hypocretin is mildly sedative and certainly not strongly stimulant. Other differences from mammalian physiology include the postulated role of hypocretin in integrating metabolic input in mice [75], as we did not find differences in locomotor activation after fasting. Environmental modulators of hypocretin release (e.g., stress, locomotion, feeding, and fasting), as measured using cerebrospinal fluid hypocretin-1, have also been shown to have differential effects between mammals, possibly reflecting ecological differences in the need for hypocretin to regulate sleep or wake under specific conditions [29]. Together with the mutant data, we therefore favor the hypothesis that hypocretin is a more minor sleep regulatory molecule in zebrafish than in mammals, with mostly sleep-promoting effects in the dark. It is also possible that in mammals, hypocretin has a dual effect on promoting sleep at night and wake during the day, thus explaining insomnia and daytime sleepiness in its pathology. In this case, only sleep-promoting projections of hypocretin would be common to zebrafish and mammals.

The differential expression pattern of the sole hypocretin receptor in zebrafish versus those in mammals may explain the mild *hcrtr^168^* insomnia phenotype and the lack of stimulatory effect of hypocretin. We have shown absence of Hcrtr on serotoninergic cells of the raphe nuclei and histaminergic cells. Moreover, unlike in a previous study [50], we were unable to find *hcrtr* expression in all diencephalic dopaminergic cells or in the locus coeruleus of 2-dpf embryos (Figure 3), a result we extended to adult brains (Figure 4). In mammals, the histaminergic tuberomammillary nucleus, the serotoninergic raphe nuclei, and the adrenergic locus coeruleus are among the densest regions containing hypocretin receptor mRNA [19]. In zebrafish, we previously noted branching of Hcrt neuron projections in the ventral area of the rhombomere 1–2 boundary [48], but believe this projection to be consistent with the strong *hcrtr* expression within rhombomere 2 rather than locus coeruleus (rhombomere 1) (Figure 3). Immunocytochemical experiments have suggested hypocretin projections to these monoaminergic systems in adult zebrafish, but they used mammalian anti-hypocretin that is cross-reactive, as reflected by a mismatch between ISH and immunocytochemical staining patterns of cell bodies [48–50]. Further, in a study using a hypocretin-GFP line, direct contact between hypocretin terminals and monoaminergic cell bodies was not evident [50], unlike in mammals [20].

Another major regulator of sleep and REM sleep in mammals is the cholinergic system, most notably through projections of the basal forebrain (regulating wake) and the pons (regulating wake and REM sleep) to the cortex and thalamus, respectively [2,24,58]. This system is also known to be excited by hypocretin and to be hypocretin-receptor-positive in mammals [24,25]. Although data in larvae were suggestive of a possible colocalization in the diencephalon and rhombencephalon (Figure 5), we found that in adults, areas believed to be equivalent to pontine and basal forebrain cholinergic cell groups in fish—the lateral part of the ventral telencephalon and the peri-locus coeruleus areas, respectively[76,77]—no ChAT transcripts were detected.

The lack of strong hypocretin input on sleep-related monoaminergic and cholinergic cell groups likely explains the weaker effect of hypocretin on wake consolidation. We have postulated that one of the key functions of hypocretin may be to extend wakefulness in the face of a mounting sleep debt [78]. In mammals, hypocretins strongly innervate the histaminergic cells of the tuberomammillary region [19,28], and the most important mechanism of action of hypocretin on wakefulness may be through the action of histamine on excitatory histamine H1 receptors [28,79]. We have recently demonstrated that the wake-promoting action of the H1 receptor is conserved in zebrafish larvae, where strong a hypnotic effect results from treatment with the H1 inverse agonist mepyramine [52]. The absence of hypocretin receptors on histaminergic cells in zebrafish is consistent with an absence of a hypersomnia phenotype in *hcrtr* mutant animals. Similarly, hypocretin innervation of the locus coeruleus in mammals has been suggested to control cataplexy and muscle atonia during REM sleep [80].

Although speculative at this stage, one pattern of expression, the presence of Hcrtr on a small population of Adra2a-positive, GABAergic cells of the anterior hypothalamic area, may be of relevance to the mediation of short sleep and nighttime sleep fragmentation in *hcrtr^168^*. Indeed, this area may be equivalent to mammalian preoptic/basal forebrain GABAergic, Adra2a-positive cell groups (including the median and ventrolateral preoptic nuclei and basal forebrain) known to be primarily active during non-REM sleep [58,81]. A loss of hypocretin receptor in this cluster would be predicted to decrease the activity of this sleep-promoting area, in turn decreasing sleep. Interestingly, the equivalent region in mammals, or at least the ventrolateral preoptic nuclei, seems mostly nonresponsive to hypocretin [58,82,83].

In conclusion, we report on the unique characteristics of sleep regulation and of the hypocretin system in zebrafish. Our data offer intriguing parallels with and surprising divergences from mammalian sleep physiology. Not only did we find that light has a profound effect on sleep, but it abolished the need for short-term homeostatic rebound. Further, in this species, hypocretin is a milder sleep modulator, possibly primarily consolidating sleep rather than wake. This is likely explained by the lack of hypocretin stimulation of monoaminergic and cholinergic systems and a proportionally stronger input on sleep-promoting systems of the hypothalamus and basal forebrain. The need for hypocretin innervation of wake-promoting structures may have evolved later, as the importance of the direct effects of light and melatonin on brain activity decreased, and the need to consolidate wake independently of light effects evolved. It is possible that, as for circadian biology, neural networks regulating behaviors (e.g., clock output networks that must vary between nocturnal and diurnal animals) are less well conserved than core genetic actors to be discovered for the regulation of sleep. Further studies in zebrafish, a poikilotherm vertebrate, together with parallel work in birds and monotremes, may help decipher how sleep regulatory network organization evolved prior to the emergence of homeothermy and the REM/non-REM dichotomy [55,84–86].

## Materials and Methods

### Animals.

Young adult (6 mo) wild-type zebrafish (Scientific Hatcheries, http://www.scientifichatcheries.com/) were used for the sleep characterization. Zebrafish were raised and maintained in Marine Biotech (http://www.marinebiotech.com/) *Zmod* systems (28.5 °C, pH 7.0, conductivity = 500 μS) in a 14 h: 10 h light/dark cycle. The hypocretin receptor knockout *hcrtr^168^* (originally named hu2098) was identified through genomic screening of the *hcrtr* locus in F1 ethylnitrosurea-mutagenized animals (TILLING) as part of the ZF-MODELS Integrated Project in the Sixth Framework Program (http://www.zf-models.org/). The mutation at codon 168 results in an arginine (AGA) to stop (TGA) alteration. Heterozygous animals were obtained and out-crossed twice to wild-type (Scientific Hatcheries). Wild-type, heterozygous, and homozygous siblings (larvae and 6 mo old) were used for the primary comparison of the three genotypes. As no significant differences were observed across all wild-types, the wild-type categories were pooled in some analyses.

### AFSRS.

The entire AFSRS is kept insulated from light in a sealed black box. Under lights-on conditions, illuminance is fixed at 150 lux, as measured on the water surface (Tensor VisionMax, 13-W fluorescent light, Tensor Corporation). Fish recording chambers consist of four individual cells (400 ml each). Adult fish are freely swimming (three dimensional movement) in vertically and horizontally stacked chambers. System water is circulated at 60 ml/min (Pondmaster magnetic drive utility pump, Danner Manufacturing, http://www.dannermfg.com/) and maintained under controlled conditions (temperature = 28.5 ± 1 °C, conductivity = 500 μS, pH 7.0). Filtration (Penguin 100, Bio-Wheel power filter, AQUARIA, Marineland, http://www.marineland.com/) and heating (Ebo-Jager 100 Watt, Eheim, http://www.eheim.de/) are also provided. Infrared backlighting (C47192–880, Advanced Illumination, http://www.advancedillumination.com/) is provided during recording. Video images (320 pixels × 240 pixels) are obtained using Sony (http://www.sony.com/) DCR-HC85 or Hamamatsu (http://www.hamamatsu.com/) Photonics C2741–60 cameras equipped with an infrared filter (B+W 093, Hamamatsu). Images are captured with a frame grabber (Dazzle Creator80, Pinnacle Systems, http://www.pinnaclesys.com/), recorded (VirtualDub for video image, http://www.virtualdub.org/), and processed using a modified algorithm of SleepWatch provided by Irina Zhdanova (Boston University). In our recording conditions, one pixel is equal to 0.6 mm (100 pixels for one cell). Data were further analyzed using Mat Lab (MathWorks, http://www.mathworks.com/).

To quantify fish activity, digital infrared images are recorded (30 frames/s) through backlit chambers, and processed using software extensively modified from SleepWatch. This software first identifies the fish and its barycenter. Successive barycenters are calculated every 1/8th second, with difference in position representing the distance traveled. Total pixels traveled each second is also calculated, representing an activity measurement expressed in pixels/second. Based on video observation, a value below 6 pixels/s was considered as background and reflected an immobile or slightly drifting floating fish.

For the electrical stimulation experiments, recording chambers were fitted with stainless steel sidewalls. Stimuli were generated using a NI PCI-6723 (National Instruments, http://www.ni.com/) controlled by LabView software (National Instruments). Water conductivity was fixed at 550 μS. For these experiments, distance traveled and the total surface of the fish image (pixels) were calculated frame by frame at a rate of 30 frame/s. Responses were blindly analyzed in video clips both computationally (calculation of change in velocity, or change in fish image surface, reflecting change in direction of movement) and through visual scoring of behavioral response. Responses were scored as present or absent.

### Icv injections.

Mammalian hypocretin-1 was obtained through Phoenix Pharmaceutical (http://www.phoenixpharm.com/). Mature zebrafish hypocretin-1 and −2 were synthesized from predicted sequence by Synthetic Biomolecules (http://www.syntheticbiomolecules.com/). Adult zebrafish skulls were drilled in the midline at the telencephalon–diencephalon border 5 h prior icv injection. After this surgery, fish recovered in water at standard temperature and conditions. One minute before injection, fish were anesthetized using 0.15 mg/ml tricaine (note that at this concentration fish recover quickly, within a few minutes, when transferred to aquarium water). Then 0.5 μl of hypocretin peptide solution (concentrations: 0.28 and 2.8 nmol/μl) or saline buffer was injected into the diencephalic and tectal ventricals through this drill hole with a micromanipulator and a glass capillary needle connected to a microinjector (PLI 90, Harvard Apparatus, http://www.harvardapparatus.com/). Fish were then released in the recording chambers, and monitoring was initiated 5–10 min after injection. Injection quality was checked by coinjecting hypocretin with a rhodamine dye and by inspection of dissected injected brains the following day. Of note, none of the fish died or appeared abnormal in the following days to weeks after injections.

### Statistical analyses.

Data are reported as means ± standard error of the mean or percent, where most appropriate. For signal detection analysis, ROC and quantitative ROC analysis were performed as described in Kraemer [56] and above. Comparisions of arousal threshold reactivity ratios between active and asleep animals were performed using Chi^2^. To examine the effect of sleep deprivation, ANOVAs with grouping factors were used, followed by *t*-tests to examine post hoc effects. For icv injections, repeated measure MANOVAs with increasing doses of hypocretin-1 as grouping factors were used, with log 10 transformation of hypocretin dose.

### Identification and cloning of teleost hypocretin receptor genes.

A fragment of the *hcrtr* gene of Tetraodon nigroviridis was identified through a BLAST search of the GenBank Genome Survey Sequences Database (http://www.ncbi.nlm.nih.gov/dbGSS/; accession AL310684). The corresponding clone (52F08) was obtained from Genoscope (http://www.cns.fr/), and the exons of the gene were identified through sequencing. *hcrtr*-containing BAC clones were first identified using the Genome System BAC library (Incyte, http://www.incyte.com/) and a Tetraodon hcrtr exon 2 probe at low stringency 48 °C (20B8, 20A23, 31L8, 59B13, and 128J8). No evidence for locus heterogeneity was evident after fingerprinting and Southern blotting. Further screening using a *hcrtr*-specific probe identified the following clones from the CHORI 211 BAC library (http://bacpac.chori.org/): L80L23, 16D22, 103I5, and 99C14. Zebrafish exons were identified through sequencing of subclones, and confirmed through reverse transcriptase PCR. The 5′ end of the gene was characterized through 5′ RACE (Generacer, Invitrogen, http://www.invitrogen.com/).

### ISH.

Whole mount ISH was performed as previously described [87]. In some cases, stained embryos were embedded in 1% agarose (Invitrogen) and cut with a vibratome (series 1000, Sectioning System) to verify colocalization or to allow better visibility of expression patterns. ISH was also performed on adult brain tissue. In this case, brains were excised and fixed in 8% PFA for 48 h, sectioned by vibratome, and stained as free-floating slices according to the procedure above. The following genes/probes were used: *adra2a, chat, dat, dbh, ddc, gad65, gad67, glyt2, hcrtr, hdc, npy, pomca, pyy, tph1, tph2, th, vglut1, vglut2a,* and *vglut2b*.

## Supporting Information

Figure S1 Effect of Constant Light (11 d) on Mean Activity Levels and Sleep Time Mean activity levels (per 2 h, top, *n =* 8) and sleep time (percent time per 2 h, bottom, *n =* 8). For details on the sleep recording system, see legend to Figure 1. Note that in this experiment fish were fed normally. Shaded area indicates normal light/dark conditions, followed by constant light. Note that activity decreases and sleep time gradually increases over the last 4 d.
(2.7 MB DOC)

Figure S2 Habituation of Fish of Three Genotypes When Moved from Their Usual Aquaria to the Recording Chamber Note diurnal fluctuation of activity of decreasing amplitude every successive day. Importantly, the diminution is parallel and identical across the three genotypes, indicating that the *hcrtr^168^* mutants are not more disturbed or activated than wild-types in the presence of a novel stressful environment. This result suggests that sleep fragmentation in *hcrtr^168^* is not the result of increased stress.
(1.1 MB DOC)

Figure S3 Food Satiety Studies in the Three Genotypes Fish of the three genotypes were studied under their regular environment but separated in individual aquaria. At the usual feeding time, a batch of 200 brine shrimp (233/100 μl) was delivered in each aquarium. After complete consumption of the brine shrimp, another batch was delivered every 5 min multiple times until it was noted that the fish stopped eating in the presence of brine shrimps. Note that all three genotypes achieved satiety at the same level, indicating that *hcrtr^168^* homozygous mutants have food intake abilities similar to those of wild-types and heterozygotes. This experiment was also performed in fish after 6 d of food restriction, and no difference was noted in six wild-type siblings, two heterozygotes, and six *hcrtr^168^* homozygous mutants (data not shown).
(1.2 MB DOC)

Figure S4 Activation of Fish by Food Restriction and Activity Resulting from Feeding After habituation and regular feeding in the recording apparatus, fish of the three genotypes were studied for 6 d without administration of food. Left: baseline activity at 3 p.m. on day 1. Right: baseline activity after 6 d of food deprivation and response of the three genotypes during food consumption (arrow, 3 p.m. under light condition). Note that the three genotypes reacted similarly. Further, baseline activity (day 1) did not increase with food restriction (day 6).
(1.6 MB DOC)

Video S1 Example of a Sleep Period, as Observed in an Adult Fish during the Daytime Note high activity level prior to sleep period. In a second phase, the fish slows down and its caudal fin droops occasionally. It then moves to the bottom of tank, caudal fin down, and rests for approximately 1 min, before resuming activity**.**
(8.3 MB MOV)

Video S2 Increased Arousal Threshold during Rest Fish are stimulated with a 10-ms pulse of 1 V (onset of stimulation is shown by an infrared light dot on the left side of video). Note immediate reactivity of active fish (left), but not of immobile fish (right).
(729 KB MOV)

Video S3 Sleep Deprivation and Reactivity of Fish at the Onset of the Sleep Deprivation Procedure The video was recorded 10 min after the start of the sleep deprivation procedure. The fish in the upper chamber is submitted to an electrical stimulation (at infrared light) every time sleep is achieved (activity < 6 pixels/s for at least 6 s). The fish in the bottom chamber (yoked control) is stimulated at the same time, whether awake or asleep. Note reactivity of both fish.
(419 KB MOV)

Video S4 Sleep Deprivation and Reactivity of Fish at the End of the Sleep Deprivation Procedure The fish in the upper chamber has been submitted to 5 h 50 min of sleep deprivation (video recorded 10 min prior to usual light on). Electrical (2–6 V) stimulation (at infrared light) is applied every time sleep is achieved (activity < 6 pixels/s for at least 6 s). Note that the sleep-deprived fish remains immobile in the middle of the chamber in spite of repetitive stimuli of increasing intensity. Yoked control (lower chamber) and sleep-deprived fish are released 10 min thereafter to monitor sleep rebound.
(5.5 MB MOV)

Video S5 AFSRS Images are recorded through backlit chambers using an infrared video camera (30 frames/s, top). Video images are stored in a computer using the VirtualDub software (upper image) and independently processed using software extensively modified from SleepWatch. This software identifies the fish (bottom left), a barycenter is calculated, and distance is traveled measured between successive barycenters every 1/8th second, resulting in a total pixels traveled measurement every second (bottom right). This measure is considered an activity measurement, as expressed in pixels/second.
(2.2 MB MOV)

### Accession Numbers

The NCBI (http://www.ncbi.nlm.nih.gov/) accession number for zebrafish *hcrtr* is EF150847. The NCBI (http://www.ncbi.nlm.nih.gov/) or Ensembl (http://www.ensembl.org/index.html) accession numbers for the genes/probes used for ISH are *adra2a* (NM_207637), *chat* (ENSDARG00000015854), *dat* (AF318177), *dbh* (ENSDARG00000058086), *ddc* (ENSDARG00000016494), *gad65* (AF017265), *gad67* (AB183390), *glyt2* (Ab183389), *hcrtr* (EF150847), *hdc* (EF150846), *npy* (NM_001007218), *pomca* (ENSDARG00000043135), *pyy* (NM_131016), *tph1* (NM_178306), *tph2* (NM_214795), *th* (AF075384), *vglut1* (AB183385), *vglut2a* (AB1386), and *vglut2b* (AB183387).

## Abbreviations

AFSRS: adult fish sleep recording system
BAC: bacterial artificial chromosome
dpf: day(s) postfertilization
icv: intracerebroventricular(ly)
ISH: in situ hybridization
REM: rapid eye movement
ROC: receiver operator curve
SE: sensitivity
SP: specificity
