# Supplementary material for: Characterization of Sleep in Zebrafish and Insomnia in Hypocretin Receptor Mutants
Source: PLoS Biol. 2007 Oct 16;5(10):e277. doi: 10.1371/journal.pbio.0050277 (PMC2020497; doi:10.1371/journal.pbio.0050277)
Supplement: Supplementary file 1 [file pbio.0050277.sg001.doc]

**Supplementary Figure S1.** Effect of constant light on mean activity levels and sleep time.
