# Supplementary material for: Characterization of Sleep in Zebrafish and Insomnia in Hypocretin Receptor Mutants
Source: PLoS Biol. 2007 Oct 16;5(10):e277. doi: 10.1371/journal.pbio.0050277 (PMC2020497; doi:10.1371/journal.pbio.0050277)
Supplement: Supplementary file 2 [file pbio.0050277.sg002.doc]

**Supplementary Figure S2.** Habituation of fish of three genotypes when moved from their usual Aquaria to the recording chamber.
